# Morphology and molecular phylogeny of a new ciliate *Anteholosticha nanjiensis* sp. nov. (Ciliophora, Hypotrichia) collected from the coastal intertidal zone of the East China Sea

**DOI:** 10.3897/zookeys.1273.184181

**Published:** 2026-03-16

**Authors:** Xuetong Zhao, Zehao Wu, Yaohan Jiang, Xiangrui Chen

**Affiliations:** 1 Laboratory of Protozoans in Coastal Water Environments, School of Marine Sciences, Ningbo University, Ningbo 315800, China School of Marine Sciences, Ningbo University Ningbo China

**Keywords:** hypotrichs, new species, SSU rDNA sequence, taxonomy

## Abstract

A new hypotrich ciliate, *Anteholosticha nanjiensis***sp. nov**., was isolated from a sandy beach in Wenzhou, China. Its morphology was investigated using detailed live observation and protargol staining, and its phylogenetic position was assessed through small subunit ribosomal DNA (SSU rDNA) sequence analyses. The new species is characterized as follows: size in vivo 90–115 × 40–50 µm; body shape elliptical and dorsoventrally flattened; 2–4 globular macronuclear nodules; 8–14 midventral cirral pairs; colourless cortical granules (about 0.5 µm in diameter) arranged in short longitudinal rows. Phylogenetic analyses robustly placed *A. nanjiensis***sp. nov**. within a clade containing *A. pseudomonilata* Li et al., 2011a and *A. marimonilata* Xu et al., 2011. However, it differs from these congeners by having 13 and 14 nucleotides in its SSU rDNA sequence, respectively, supporting its status as a distinct species. Morphologically, *A. nanjiensis***sp. nov**. can be distinguished from related species by its body size, number of macronuclear nodules, transverse cirri count, and cortical granulation distribution. This study provides comprehensive morphological and molecular data establishing foundational references for resolving the apparent polyphyly of *Anteholosticha*. While full resolution may require integration of additional evidence (e.g. morphogenetic data or TEM ultrastructure), our dataset delivers essential comparative frameworks for future taxonomic revisions.

## Introduction

Ciliates (Ciliophora Doflein, 1901) are globally distributed, morphologically diverse, and highly specialized microbial eukaryotes that serve as flagship models for ecological, evolutionary, and cell biological research (e.g. Lynn 2008; Chi et al. 2024; Li et al. 2024; Wu et al. 2024; Gao et al. 2025; Jiang et al. 2025; Liu et al. 2025; Ma et al. 2025). Among them, the subclass Hypotrichia Stein, 1859 represents one of the most morphologically complex and taxonomically challenging groups. Persistent discrepancies between morphology-based classifications and molecular phylogenies have rendered their systematics problematic (Song et al. 2009; Foissner 2016; Omar et al. 2025; Han et al. 2025). This confusion largely stems from insufficient characterization using modern integrative approaches, hindering our understanding of their evolutionary relationships. Despite these challenges, hypotrichs exhibit high diversity and significant evolutionary importance.

Within the hypotrichs, urostylids represent a highly diverse and morphologically complex group. They are primarily characterized by a prominent midventral complex and a dorsal ciliature composed of bipolar kineties. Approximately 200 species have been described to date, inhabiting diverse ecosystems worldwide (Berger 2006, 2008, 2011; Luo et al. 2021; Song et al. 2024; Gao et al. 2025; Zhao et al. 2025). Berger (2003) established the genus *Anteholosticha* to accommodate species previously classified under *Holosticha* sensu Borror (1972) that lack defining autapomorphies (e.g. anterior end of left marginal cirral row curved rightwards, bipartite adoral zone, buccal cirrus distinctly ahead of paroral membrane). However, subsequent molecular phylogenetic studies have consistently revealed the polyphyly of *Anteholosticha* (Berger 2006, 2008; Park et al. 2012, 2013; Huang et al. 2014; Zhao et al. 2015; Chen et al. 2018, 2020; Jung et al. 2021; Song et al. 2021). Based on a phylogenetic analysis of three genes, Huang et al. (2014) transferred three species of *Anteholosticha*, namely *A. scutellum* (Cohn, 1866) Berger, 2003, *A. petzi* Shao et al., 2011 and *A. warreni* (Song & Wilbert, 1997) Berger, 2003, each of which has its transverse cirri arranged in a roughly U-shaped pseudorow, to the newly established genus *Arcuseries*. Despite over 40 nominal species assigned to *Anteholosticha*, many remain poorly documented, frequently lacking detailed morphological descriptions and molecular data (Berger 2006; Fan et al. 2016; Chen et al. 2018, 2020). These critical knowledge gaps significantly impede systematic and taxonomic progress within the genus.

In the present study, a new species, *Anteholosticha nanjiensis* sp. nov., was isolated from a sandy beach in Wenzhou, China. Its morphology was observed using both live observation and protargol staining. The taxonomy and phylogenetic position of this species were assessed based on detailed morphological characterization and SSU rDNA sequence analysis.

## Materials and methods

### Sample collection, observation, and identification

**Figure 1.**
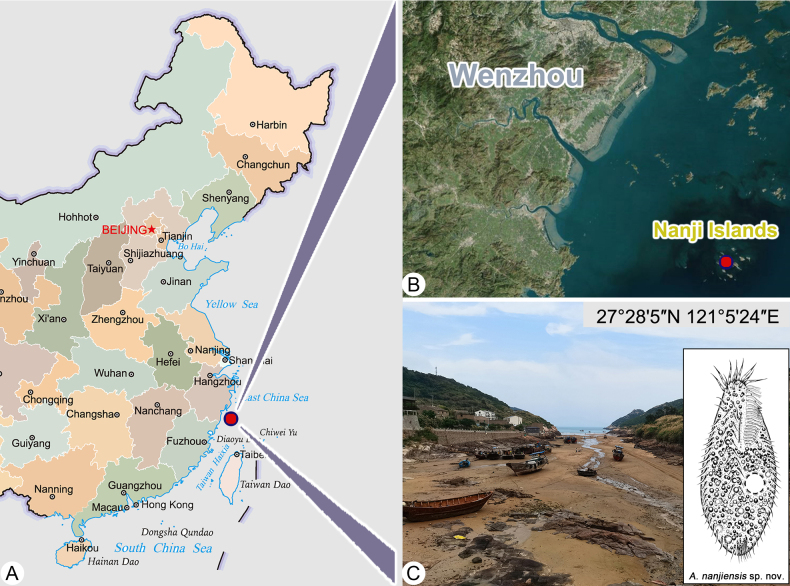
Maps showing the sampling locations in Wenzhou and photographs of the sampling sites. **A**. Part of the map of China; **B**. The portion of the map of China shows the location of sampling sites in Wenzhou (colored circle and words); **C**. Sampling site of *Anteholosticha nanjiensis* sp. nov.

Samples were collected from a site in Wenzhou, Zhejiang Province, China (Fig. 1A–C). *Anteholosticha nanjiensis* sp. nov. was isolated from a sandy beach sample (27°28'5"N, 121°5'24"E) on 17 November 2022. At the time of collection, the water temperature was about 24 °C and the water salinity was 25‰. Clonal cultures were maintained in Petri dishes at room temperature for several days using filtered in situ water. To promote bacterial growth as a food source for the ciliates, rice grains or wheat grass juice was added. Although long-term culture could not be maintained, sufficient individuals from the species were successfully obtained within the one-week cultivation period for detailed morphological and phylogenetic analyses.

Living cells were observed using bright field and differential interference contrast (DIC) microscopy (Leica DM2500, Germany) at 400–1,000× magnification. Protargol staining, following the method of Wilbert (1975), was used to reveal the ciliature and nuclear apparatus. Counts, measurements, and stained specimen drawings were performed at 1,000× magnification. Terminology is mainly according to Berger (2006), and the systematic classification follows by Lynn (2008) and Luo et al. (2015).

### DNA extraction, PCR amplification, and gene sequencing

Single cells were isolated from Petri dishes via micropipette. Each cell was washed 3–5 times with filtered in situ water, followed by two washes with sterile distilled water, before transfer to a sterile 1.5 ml microfuge tube for DNA extraction. Three to five parallel molecular samples from each population will be prepared. Genomic DNA was extracted using the DNeasy Blood & Tissue Kit (Qiagen, Hilden, Germany), following the manufacturer’s instructions. The PCR primers for 18S rDNA amplification were 82S-F (5'-GAA ACT GCG AAT GGC TC-3') (Jerome et al. 1996) and 5.8S-R (5'-TAC TGA TAT GCT TAA GTT CAG CGG-3') (Gao et al. 2012). To minimize amplification errors, we employed the Q5 Hot Start High-Fidelity DNA Polymerase (New England BioLabs, USA). The PCR conditions were as follows: (1) initial denaturation at 98 °C for 30 s; (2) 35 cycles of denaturation at 98 °C for 10 s, annealing at 56 °C for 20 s, and extension at 72 °C for 100 s, and (3) a final extension at 72 °C for 5 min.

Sequencing was performed bidirectionally at Tsingke Biological Technology Company (Hangzhou, China), using the primers 82S-F, 5.8S-R, 900F (5'-CGA TCA GAT ACC GTC CTA GT-3'), and 900R (5'-ACT AGG ACG GTA TCT GAT CG-3') (Wang et al. 2017). SeqMan v. 5.0 (DNAStar) was used to assemble the contigs, covering the partial 18S rDNA and the ITS1-5.8S rDNA-ITS2 region. Primer 18S-R (5'-TGA TCC TTC TGC AGG TTC ACC TAC-3') (Medlin et al. 1988) was used to remove the ITS1-5.8S rDNA-ITS2 fragment.

### Phylogenetic analyses

Phylogenetic analyses were performed on 78 SSU rDNA sequences, including one newly acquired sequence and 77 sequences downloaded from the National Center for Biotechnology Information (NCBI). The GenBank accession numbers are provided in the phylogenetic tree (Fig. 4). Outgroup taxa included four Euplotida species (*Diophrys scutum* (Dujardin, 1841) Kahl, 1932 JF694040, *Uronychia multicirrus* Song, 1997 EU267929, *Apodiophrys ovalis* Jiang & Song, 2010 GU477634, and *Paradiophrys zhangi* Jiang et al., 2011 FJ870076). The sequences were aligned, using the GUIDANCE2 Server (http://guidance.tau.ac.il) with default parameters (Penn et al. 2010) and manually edited in BioEdit v. 7.0.5.3 (Hall, 1999). Maximum-likelihood (ML) analysis was conducted on the CIPRES Science Gateway (http://www.phylo.org/portal2), using RAxML-HPC2 on XSEDE v. 8.2.12 (Stamatakis 2014) with the GTR + I + г model. The support for the ML tree was tested using 1,000 bootstrap replicates. Bayesian-inference (BI) analysis was performed on CIPRES Science Gateway with MrBayes on XSEDE v. 3.2.6 (Ronquist and Huelsenbeck 2003) using the GTR + I + г model selected by MrModeltest v. 2.2 (Nylander 2004). The BI analysis was conducted across 10^6^ generations, with trees sampled every 100^th^ generation. The first 2,500 trees were discarded as burn-in. Tree topologies were visualized via MEGA v. 7.0 (Kumar et al. 2016) and TreeView v. 1.6.6 (Page 1996).

## Results

### Taxonomy

**Subclass Hypotrichia Stein, 1859**

**Order Urostylida Jankowski, 1979**

**Family Urostylidae Bütschli, 1889**

**Genus *Anteholosticha* Berger, 2003**

#### Anteholosticha nanjiensis sp. nov.

Taxon classificationAnimaliaUrostylidaHolostichidae

71E5E9E7-24E9-50FA-923B-78F088FD53E8

https://zoobank.org/6DA2168B-704F-47F1-8645-DA78BD124B78

**Figure 2.**
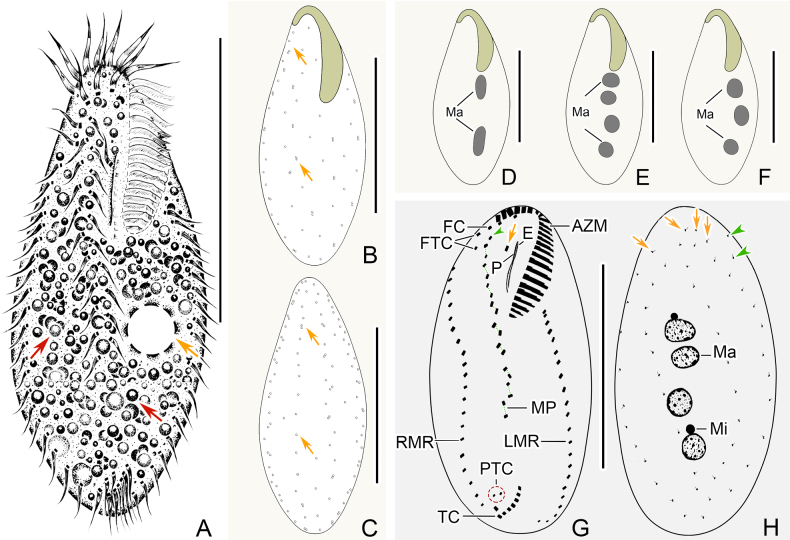
*Anteholosticha nanjiensis* sp. nov. **A–F**. In life; **G, H**. Following protargol staining. **A**. Ventral view of a representative specimen; orange arrow marks the contractile vacuole, and the red arrows point to the cytoplasm granules; **B, C**. Ventral and dorsal views of the same specimen; arrows show the distribution of the cortical granules; **D–F**. Ventral views of different specimens indicate different number of macronuclei; **G**. Holotype, ventral view; arrow marks the buccal cirrus, and arrowhead indicates the parabuccal cirrus; **H**. Holotype, dorsal view; arrows mark the dorsal kineties, and arrowheads point to the basal body pairs at the anterior end of right marginal row. Abbreviations: AZM, adoral zone of membranelles; E, endoral; FC, frontal cirri; FT, frontoterminal cirri; LMR, left marginal row; Ma, macronuclear nodules; Mi, micronuclei; MP, midventral pair; MVR, midventral cirral row; P, paroral; PTC, pretransverse ventral cirri; RMR, right marginal row; TC, transverse cirri. Scale bars: 60 μm.

**Figure 3.**
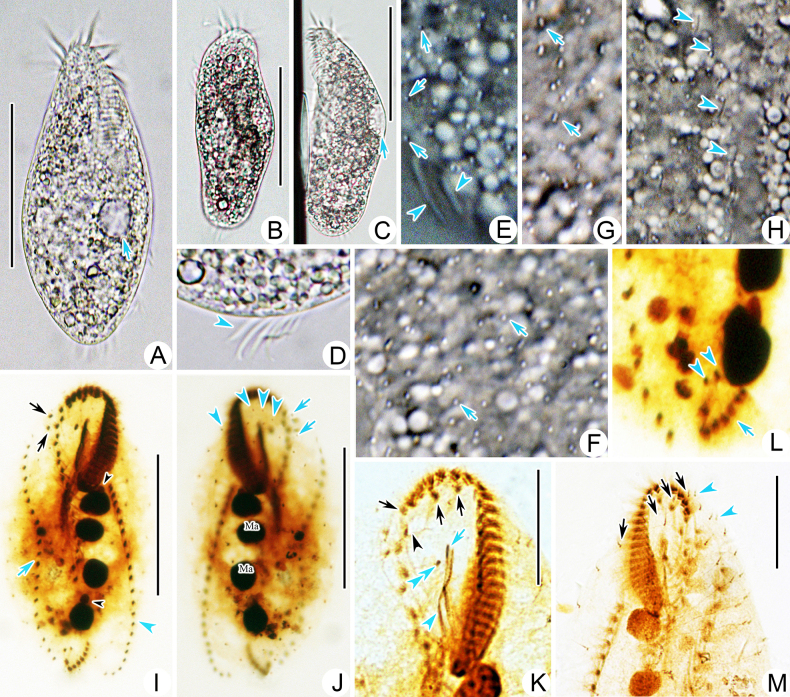
Photomicrographs of *Anteholosticha nanjiensis* sp. nov. **A–G**. In life; **H–L**. Following protargol staining. **A**. Ventral view of represent individual to show the body shape; arrow marks the contractile vacuole; **B**. Ventral views show the different individual; **C**. Ventrolateral view; arrow indicates the contractile vacuole; **D**. Ventral view; arrow marks the transverse cirri; **E**. Lateral to ventral views show the cortical granules; arrows mark the cortical granules, and arrowheads point to the transverse cirri; **F**. Ventral view; arrows show the distribution of the cortical granules; **G**. Dorsal view; arrows show the distribution of the cortical granules; **H**. Dorsal view; arrowheads indicate the dorsal kineties; **I**. Holotype, ventral view; black arrow denotes the frontoterminal cirri, blue arrow points to the right marginal row, blue arrowhead points to the left marginal row, and black arrowheads mark the micronuclei; **J**. Holotype, dorsal view; blue arrowheads mark the dorsal kineties, and blue arrows point to the basal body pairs at the anterior end of right marginal row; **K**. Anterior ventral cell portion; black arrows denote the frontal cirri, black arrowhead points to the parabuccal cirrus, blue arrow marks the paroral, blue arrowhead indicates the endoral, and blue double arrowhead points to the buccal cirrus; **L**. The posterior dorsal cell portion; arrow shows the transverse cirri, and arrowheads point to the pretransverse ventral cirri; **M**. Dorsal view; arrows mark the dorsal kineties, and arrowheads point to the basal body pairs at the anterior end of right marginal row. Abbreviations: Ma, macronuclear nodules. Scale bars: 60 μm **(A–C, I, J)**; 30 μm **(K, M)**.

Figs 2A–H, 3A–L, 3Table 1

##### Diagnosis.

Body size in vivo 90–115 × 40–50 μm; anterior portion slightly narrowed, posterior end broadly rounded. Cortical granules colourless, spherical, about 0.5 μm in diameter, arranged in more or less longitudinal short rows on whole cortex. Macronuclear nodules 2–4. Adoral zone of membranelles composed of 26–37 membranelles. Two frontoterminal cirri. Midventral cirral pairs 8–14. One right and one left marginal cirral row consisting of 19–28 and 18–28 cirri, respectively. Four bipolar dorsal kineties composed of 10–18, 10–16, 10–17, and 11–17 dikinetids, respectively.

##### Etymology.

The species name nanjiensis refers to Nanji Island, where the new taxon had been discovered.

##### Type materials.

Five slides with protargol staining specimens have been deposited in the ciliate specimen repositories, including one slide (registration number WZH-20221117-01) with the holotype specimen deposited in the Laboratory of Protozoology, Ocean University of China (OUC) and four slides (registration numbers WZH-20221117-02, 03, 04, 05) with paratype specimens deposited in Laboratory of Protozoans in Coastal Water Environments, Ningbo University.

##### Morphological description.

Body size in vivo 90–115 × 40–50 μm and 93–160 × 38–73 μm after protargol staining (Figs 2A, 2G, 2H, 3A, 3I, 3J). Body shape slightly variable and elliptic; anterior portion slightly narrowed and forming a slightly cephalized appearance, while posterior end broadly rounded (Figs 2A, 3A, 3B). Dorsoventrally flattened about 2:1. Cytoplasm colourless to light grey, containing some lipid droplets, food vacuoles, and some irregular crystals (Figs 2A, 3A, 3B). One contractile vacuole about 11–13 μm across during diastole, positioned in mid-body near left margin (Figs 2A, 3A, 3C). Cortical granules colourless, spherical, about 0.5 μm in diameter, arranged in more or less longitudinal short rows on whole cortex with some single ones sparsely distributed throughout the cell surface (Figs 2B, 2C, 3E–G). Macronuclear nodules 2–4 (usually 4), globular to ellipsoidal, serially distributed in mid-portion of body left of midline which can be easily observed under moderate to high magnification in vivo, with nodule sizes about 7–19 × 6–11 μm (after protargol staining) (Figs 2D–F, 2H, 3I). Micronuclei 1–2, ovoid, located separately among macronuclear nodules, 6 μm in diameter (after protargol staining) (Figs 2H, 3I). Body apparently flexible, folded or twisted when crawling on bottom of Petri dish or drilling through debris.

Adoral zone of membranelles (AZM) occupying 25–39% (on average 31%) of body length after protargol staining, composed of 26–37 membranelles, with cilia up to 9–11 μm long in vivo (Figs 2A, 2G, 3A, 3I, 3Table 1). Paroral and endoral membrane almost the same length, distinctly intersecting each other and terminating anteriorly in upper 1/3 of buccal field (Figs 2G, 3I, 3K). Cirral pattern shown in Figs 2, 3. Three slightly enlarged frontal cirri lying in frontal area (Figs 2G, 3I). Single buccal cirrus situated on the right side of the anterior end of the undulating membranes (Figs 2G, 3I, 3K). Two frontoterminal cirri near and right of distal end of adoral zone of membranelles (Figs 2G, 3I, 3K). Cirri 6–8, relatively transverse, and arranged in J-shaped row (Figs 2G, 3I, 3L). Always two pretransverse ventral cirri located ahead of the right transverse cirrus (Figs 2G, 3L). Midventral complex only composed of 8–14 pairs of midventral cirri arranged in a typical zigzag pattern, continuing with frontal cirri and extending to the rear 1/3 of the body length (Figs 2G, 3I). One right and one left marginal cirral row comprising 19–28 and 18–28 cirri, respectively (Figs 2G, 3I).

Invariably, four complete bipolar dorsal kineties, composed of 10–18, 10–16, 10–17, and 11–17 dikinetids, respectively; One or two bristles recognized ahead of right marginal cirral row in eightof 20 individuals examined (Figs 2H, 3J, 3M, 3Table 1). Dorsal cilia about 3 μm long in vivo (Fig. 3H).

### SSU rDNA sequences and phylogenetic analyses

**Figure 4.**
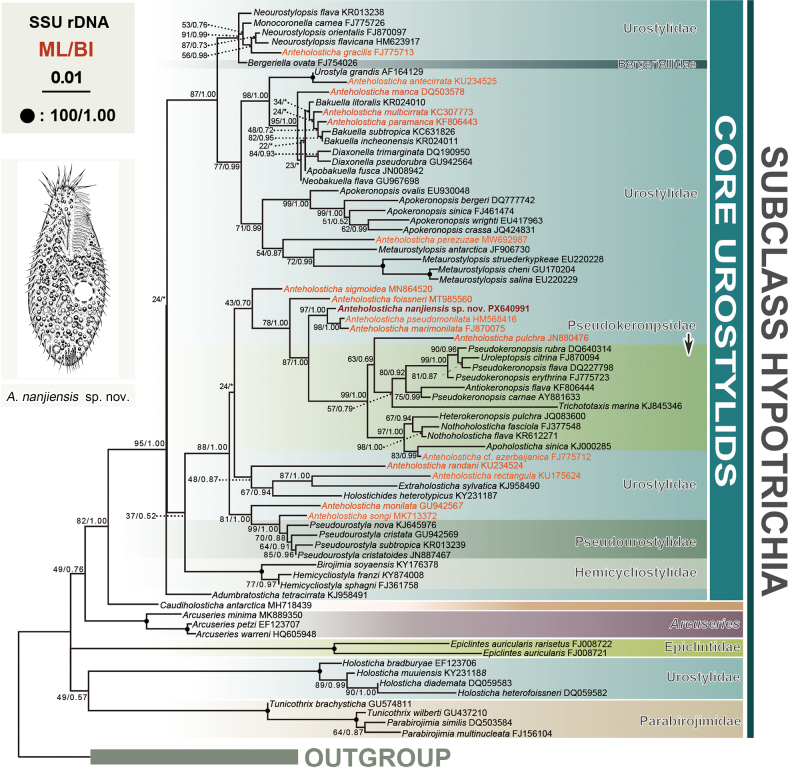
Maximum-likelihood (ML) tree based on SSU rDNA sequence data. The newly sequenced *Anteholosticha nanjiensis* sp. nov. is indicated in bold red. The orange text indicates sequences belonging to the genus *Anteholosticha*. Numbers at nodes indicate the ML bootstrap values and the posterior probabilities of Bayesian analysis (BI). Solid circles represent full support from both algorithms. The scale bar corresponds to one substitution per 100 nucleotide positions.

The newly obtained SSU rDNA sequence of *Anteholosticha nanjiensis* sp. nov. has been deposited in the GenBank database under accession number PX640991. The sequence length is 1,632 bp with a G + C content of 45.28%. Phylogenetic trees reconstructed using both ML and BI methods yielded nearly identical topologies. Consequently, only the ML tree is presented, with support values from both methods indicated (Fig. 4).

The new sequence was grouped within a robustly supported clade (ML/BI, 97%/1.00) containing two *Anteholosticha* species: *Anteholosticha pseudomonilata* (HM568416), and *A. marimonilata* (FJ870075). This sister cluster groups with a clade comprising two *Anteholosticha* species (*A. pulchra*JN880476 and *A. cf. azerbaijanica*FJ775712), six Pseudourostylidae spp., and five Pseudokeronopsidae spp. with moderate support (ML/BI, 87%/1.00). The type species, *A. monilata*, showed a sister relationship to the clade containing *A. songi* and *Pseudourostyla* spp. Other *Anteholosticha* species are scattered throughout the tree as shown in Fig. 4, further confirming the non-monophyly of the genus.

Sequence comparisons between *Anteholosticha nanjiensis* sp. nov. and these molecular related species show that *A. nanjiensis* sp. nov. differs from *A. pseudomonilata* (HM568416) by 13 nucleotide positions (99.20% sequence identity), from *A. marimonilata* (FJ870075) by 14 nucleotide positions (99.14% sequence identity), from *A. foissneri* (MT985560) by 34 nucleotide positions (97.92% sequence identity), from *A. sigmoidea* (MN864520) by 44 nucleotide positions (97.40% sequence identity), from *A. pulchra* (JN880476) by 59 nucleotide positions (96.60% sequence identity), and from *A. cf. azerbaijanica* (FJ775712) by 70 nucleotide positions (96.10% sequence identity) (Table 3).

**Table 1.** Morphometric data on *Anteholosticha nanjiensis* sp. nov. All data based on protargol-impregnated specimens. Abbreviations: CV, coefficient of variation in %; DK, dorsal kinety; M, Median; Max, maximum; Mean, arithmetic mean; Min, minimum; *n*, number of specimens investigated; no., number; SD, standard deviation.

Characteristics	Min	Max	Mean	M	SD	CV%	n
Body length (μm)	93	160	120.9	119	19.4	16.0	24
Body width (μm)	38	73	54.5	54	10.2	18.7	24
Adoral zone of membranelles, length (μm)	31	44	36.8	36	4.2	11.4	24
Adoral zone of membranelles length: body length, ratio	0.25	0.39	0.31	0.31	0.04	12.9	24
Adoral membranelles, no.	26	37	31.3	31	2.9	9.3	24
Frontal cirri, no.	3	3	3.0	3	0	0	24
Buccal cirri, no.	1	1	1.0	1	0	0	24
Frontoterminal cirri, no.	2	2	2.0	2	0	0	24
Midventral pairs, no.	8	14	9.7	9	1.9	19.6	24
Pretransverse ventral cirri, no.	2	2	2.0	2	0	0	24
Cirri in right marginal row, no.	19	28	23.7	24	2.5	10.5	24
Cirri in left marginal row, no.	18	28	22.3	23	2.8	12.6	24
Transverse cirri, no.	6	8	7.3	7	0.8	11.0	24
Dorsal kineties, no.	4	4	4.0	4	0	0	20
Dorsal kinety 1, bristles, no.	10	18	13.1	12	2.7	20.6	20
Dorsal kinety 2, bristles, no.	10	16	12.9	13	1.6	12.4	20
Dorsal kinety 3, bristles, no.	11	17	12.9	12.5	2.0	15.5	20
Dorsal kinety 4, bristles, no.	11	17	14.7	13	1.8	12.2	20
Dorsal bristles ahead of right marginal row, number	0	2	0.8	0	1.0	125.0	20
Macronuclear nodule, no.	2	4	3.8	4	0.5	14.2	24
Macronuclear nodule (anteriormost), length (μm)	7	19	10.2	9	3.1	30.7	24
Macronuclear nodule (anteriormost), width (μm)	5	11	8.1	8	1.2	14.3	24
Micronuclei, no.	1	2	1.3	1	0.5	38.5	7

**Table 2.** Comparison of morphological features of *Anteholosticha nanjiensis* sp. nov. with those of other *Anteholosticha* species.

Characters	*A. foissneri*	*A. marimonilata*	*A. pseudomonilata*	*A. sigmoidea*	*A. nanjiensis* sp. nov.
Body length in vivo (μm)	105–155	80–160	110–140	140^a^	90–115
Macronuclear nodules, no.	5–11	4–9	8–12	5–9	2–4
Adoral zone of membranelles length, portion (%)	34–43%	About 35%	30–42%	25–33%	25–39%
Adoral membranelles, no.	32–45	33–43	29–41	25–28	26–37
Cortical granules	yellowish	two types, colorless	colorless	colorless	colorless
Collecting canals	Absent	Absent	Present	Absent	Absent
Parabuccal cirri, no.	1	1	—	—	1
Buccal cirri, no.	1	1	1	1	1
Frontal cirri, no.	3	3	3	3	3
Frontoterminal cirri, no.	2	2	2	2	2
Midventral pairs, no.	10–13	12–17	10–16	12–18 (left row) 12–18 (right row)	8–14
Right marginal cirri, no.	27–41	26–35	24–34	22–33	19–28
Left marginal cirri, no.	27–40	23–36	23–33	24–35	18–28
Transverse cirri, no.	7–10	10–13	8–11	4–6^b^	6–8
Pretransverse cirri, no.	1–2	1–3	2	—	2
Dorsal kineties, no.	4–5	4–5	4	4	4
Habitat	Brackish water	Marine	Brackish water	Terrestrial	Marine
Data source	Jung et al. (2021)	Xu et al. (2011)	Li et al. (2011a)	Foissner (1984)	Present work

^a^ On average; ^b^ Including PTC; — Data not available.

**Table 3.** Sequence similarities (below diagonal) and nucleotide differences (above diagonal) among *Anteholosticha nanjiensis* sp. nov. and six molecularly related species.

Sequences	1	2	3	4	5	6	7
*1. A. nanjiensis* sp. nov. (PX640991)		13	14	34	44	59	70
*2. A. pseudomonilata* (HM568416)	0.992		4	35	47	65	73
*3. A. marimonilata* (FJ870075)	0.991	0.997		36	47	66	74
*4. A. foissneri* (MT985560)	0.979	0.978	0.980		40	68	83
*5. *Anteholosticha sigmoidea (MN864520)	0.974	0.973	0.974	0.977		65	91
*6. Anteholosticha pulchra* (JN880476)	0.966	0.963	0.963	0.961	0.963		63
*7. *A. cf. azerbaijanica (FJ775712)	0.961	0.959	0.958	0.953	0.949	0.965	

## Discussion

### Morphological comparison of *Anteholosticha nanjiensis* sp. nov. with its congeners

*Anteholosticha* is a speciose genus with more than 40 nominal species (Berger 2003, 2006, 2008; Li et al. 2007, 2011a, 2011b; Kumar et al. 2010; Xu et al. 2011; Park et al. 2012, 2013; Fan et al. 2014, 2016; Chen et al. 2018; Jung et al. 2021). However, it is non-monophyletic and lacks defining morphological apomorphies, and most species require further study and redescription (Berger 2003, 2006; Li et al. 2009, 2011a; Fan et al. 2014; Lv et al. 2015; Zhao et al. 2015; Jung et al. 2019).

Regarding ciliary patterns, the new species is characterized by: (i) 2–4 macronuclear nodules; (ii) three enlarged frontal cirri; (iii) four dorsal kineties; and (iv) a continuous adoral zone of membranelles. Morphologically similar taxa include *Anteholosticha verugracilis* (Berger, 2005) Berger, 2006 and *A. sigmoidea* (Foissner, 1982) Berger, 2003, which should be compared with *A. nanjiensis* sp. nov. Additionally, three molecularly allied species, namely *A. pseudomonilata* Li et al., 2011a, *A. foissneri* Jung et al., 2021, and *A. marimonilata* (Kahl, 1932) Berger, 2003, were also included in the comparative analysis.

*Anteholosticha nanjiensis* sp. nov. can be separated from *A. vuxgracilis* by the body length in vivo (90–115 μm vs 80 μm), the body length:width ratio (2–3:1 vs 6:1), the relative length of the adoral zone to body length (on average 31% vs 20%), and the number of macronuclear nodules (usually 4 vs 2).

*Anteholosticha nanjiensis* sp. nov. can be separated from *A. sigmoidea* (sensu Foissner 1984) by the by the body length in vivo (90–115 μm vs 140 μm), the number of adoral zone of membranelles (26–37 vs 25–28), the number of macronuclear nodules (2–4, usually 4 vs5–9), the number of transverse cirri (6–8 vs 4–6), and the dorsal bristles in DK1 (on average 13 vs 9)

*Anteholosticha nanjiensis* sp. nov. can be separated from *A. pseudomonilata* by the number of macronuclear nodules (2–4 vs 8–12), the body size in vivo (90–115 × 40–50 μm vs 110–140 × 40–60 μm), the existence of collecting canals (absent vs present), and the number of transverse cirri (6–8 vs 8–11) (Table 2).

*Anteholosticha nanjiensis* sp. nov. can be separated from *A. foissneri* by the number of macronuclear nodules (2–4 vs 5–11), the colour of cortical granules (colourless vs yellowish), the arrangement of cortical granules (in short longitudinal rows over cortex with some single ones sparsely distributed throughout the cell surface vs along dorsal kineties and cirral rows), and the gap between posterior ends of both marginal cirral rows (distinct vs indistinct) (Table 2).

*Anteholosticha nanjiensis* sp. nov. can be separated from *A. marimonilata* by the number of macronuclear nodules (2–4 vs 4–9), the number of transverse cirri (6–8 vs 10–13), and the number of cortical granule types (1 vs 2) (Table 2).

The genetic differences in the SSU rDNA sequences support these morphological differences. The sequence similarity between *A. nanjiensis* sp. nov. and *A. pseudomonilata*, *A. marimonilata*, and *A. foissneri* are 99.20%, 99.14%, and 97.92%, respectively, with 13, 14, and 34 deviating base pairs (Table 3). Given the high conservation of SSU rDNA, the observed genetic difference is noteworthy. Consequently, we believe that the establishment of *A. nanjiensis* sp. nov. is well supported by genetic evidence.

### Phylogenetic analyses

The genus *Anteholosticha* was first established by Berger (2003), characterized by the following combination of features: three distinct frontal cirri, a zigzag midventral complex composed only of cirral pairs, transverse cirri, single left and right marginal rows, and the absence of caudal cirri. Phylogenetic relationships within *Anteholosticha* inferred from SSU rDNA sequence data are consistent with previous studies and confirm the genus is polyphyletic (Park et al. 2013; Zhao et al. 2015; Fan et al. 2016; Chen et al. 2018, 2020; Paiva 2020; Jung et al. 2021). The newly discovered *A. nanjiensis* sp. nov. clusters with *A. pseudomonilata* (HM568416) and *A. marimonilata* (FJ870075) with high support (ML/BI, 97%/1.00). However, morphologically, *A. nanjiensis* sp. nov. can be easily separated from *A. pseudomonilata* by the number of macronuclear nodules (2–4 vs 8–12), body size in vivo (90–115 × 40–50 μm vs 110–140 × 40–60 μm), and number of transverse cirri (6–8 vs 8–11) (Li et al. 2011a); *A. nanjiensis* sp. nov. can be easily separated from *A. marimonilata* by the number of macronuclear nodules (2–4 vs 4–9), number of transverse cirri (6–8 vs 10–13), and one type of cortical granules (vs two) (Xu et al. 2011). Other *Anteholosticha* species are scattered among different branches within the core urostylid assemblage.

Until robust apomorphies emerge to clarify evolutionary relationships, this polyphyly will likely persist and can only be resolved through expanded taxonomic sampling. To address this taxonomic and phylogenetic impasse, the revised classification proposed by Paiva (2020) provides a valuable framework: the newly erected order Kentrurostylida explicitly encompasses the “core urostylids”, establishing a defined focus for future comparative studies. Within this order, two suborders have been established—Simplicitergida (characterized by three dorsal bristle rows) and Hispidotergida (bearing numerous dorsal bristles). Under Paiva’s (2020) scheme, *A. nanjiensis* sp. nov. would be tentatively assigned to Hispidotergida. This study underscores the provisional nature of taxonomy and phylogeny within both bakuellid-like lineages and the current constituents of *Anteholosticha*.

In summary, the genus *Anteholosticha* constitutes a morphologically heterogeneous assemblage of species, as evidenced by variations in macronuclear nodules count (ranging from two to multiple), number of dorsal kineties, and presence/absence of cortical granules—yet molecular data remain unavailable for over half of its constituent members. The genus currently exhibits polyphyly within phylogenetic reconstructions. In the absence of unifying morphological characters among its species, future taxonomic work must prioritize establishing monophyletic clades within the family, substantiated by definitive morphological and molecular evidence.

## Supplementary Material

XML Treatment for Anteholosticha nanjiensis

## Additional information

### Use of AI

This study utilized DeepSeek solely for improving language and refining grammer.

### Author ORCIDs

Xuetong Zhao https://orcid.org/0009-0008-4371-5344

Zehao Wu https://orcid.org/0009-0003-6055-7521

Yaohan Jiang https://orcid.org/0009-0000-6803-4248

Xiangrui Chen https://orcid.org/0000-0003-4737-4694

### Data availability

All of the data that support the findings of this study are available in the main text.
